# Analysis of polyunsaturated fatty acids and the omega-6 inflammatory pathway in hepatic ischemia/re-perfusion injury

**DOI:** 10.3892/mmr.2015.3908

**Published:** 2015-06-11

**Authors:** EBRU KIRAC, FILIZ ÖZCAN, HAZAL TUZCU, GULSUM O ELPEK, MUTAY ASLAN

**Affiliations:** 1 Department of Medical Biochemistry, Faculty of Medicine, Akdeniz University, Antalya 07070, Turkey; 2 Department of Pathology, Faculty of Medicine, Akdeniz University, Antalya 07070, Turkey

**Keywords:** liver, ischemia/re-perfusion, polyunsaturated fatty acids

## Abstract

The aim of the present study was to assess omega-3 (n-3) and omega-6 (n-6) polyunsaturated fatty acids (PUFAs) in liver tissue and evaluate changes in the n-6-associated inflammatory pathway following liver ischemia/re-perfusion (IR) injury. Male Wistar rats which were allowed free access to standard rat chow were included in the study. Blood vessels supplying the median and left lateral hepatic lobes were occluded with an arterial clamp for 60 min, followed by 60 min of re-perfusion. Levels of arachidonic acid (AA, C20:4n-6), dihomo-gamma-linolenic acid (DGLA, C20:3n-6), eicosapentaenoic acid (EPA, C20:5n-3) and docosahexaenoic acid (DHA, C22:6n-3) in liver tissue were determined by an optimized multiple reaction monitoring method using ultra fast-liquid chromatography coupled with tandem mass spectrometry. Phospholipase A2 (PLA2), cyclooxygenase (COX) and prostaglandin E2 (PGE2) were measured in tissue samples to evaluate changes in the n-6 inflammatory pathway. Total histopathological score of cellular damage were significantly increased following hepatic IR injury. n-3 and n-6 PUFA levels were significantly increased in post-ischemic liver tissue compared to those in non-ischemic controls. No significant difference was observed in the AA/DHA and AA/EPA ratio in post-ischemic liver tissues compared with that in the control. Tissue activity of PLA2 and COX as well as PGE2 levels were significantly increased in post-ischemic liver tissues compared to those in non-ischemic controls. The results of the present study suggested that increased hydrolysis of fatty acids via PLA2 triggers the activity of COX and leads to increased PGE2 levels. Future studies evaluating agents which block the formation of eicosanoids derived from n-6 PUFAs may facilitate the development and application of treatment strategies in liver injury following IR.

## Introduction

Partial or total interruption of hepatic flow is often required when liver surgery is performed. This interruption of blood flow is termed as 'warm ischemia' and upon re-vascularization, when molecular oxygen is re-introduced, the organ undergoes a process called 're-perfusion injury', which causes deterioration of organ function (1). The interruption of hepatic blood flow followed by its restoration during re-perfusion clinically occurs in a number of settings, including liver transplantation, liver resection under inflow occlusion (Pringle maneuver) and hemorrhagic shock with fluid resuscitation (2,3). Although the mechanisms by which organ damage occurs in ischemia/re-perfusion (IR) injury have not been fully elucidated, ischemia results in the termination of oxidative phosphorylation and adenosine triphosphate production through aerobic respiration. Restoration of the blood flow during re-perfusion triggers the activation of kupffer cells, causing oxygen free radical formation, production of tumor necrosis factor-alpha (TNF-alpha) and interleukin-1 (IL-1) (4). Elevated levels of the pro-inflammatory cytokines TNF-alpha and IL-1 promote polymorphonuclear neutrophil recruitment and activation, which also generates reactive oxygen species (ROS) and leads to the release of proteases (5,6).

Adherence of circulating blood cells to the vascular endothelium is modulated by polyunsaturated fatty acids (PUFAs). An increase in adherence and de-granulation of neutrophils was observed when they were incubated with arachidonic acid (AA, C20:4n-6) and dihomo-gamma-linolenic acid (DGLA, C20:3n-6) (7). Likewise, the ability of PUFAs to modulate endothelial activation was shown by a study in which docosahexaenoic acid (DHA, C22:6n-3), when added to cultured endothelial cells prior to stimulation with cytokines, reduced the adhesion of monocytes and endothelial expression of vascular cell adhesion molecule-1, E-selectin and intercellular adhesion molecule-1 (8).

The human body can produce numerous fatty acids except the two essential PUFAs, linoleic acid (LA, C18:2n6) and alpha-linolenic acid (ALA, C18:3n3). Linoleic acid is the precursor of the omega-6 (n-6) series of PUFAs, while ALA is the precursor of the omega-3 (n-3) series of PUFAs. Eicosanoids derived from n-6 PUFAs, such as AA (C20:4n-6), have pro-inflammatory and immunoactive functions, whereas eicosanoids derived from n-3 PUFAs, such as eicosapentaenoic acid (EPA, C20:5n-3), have anti-inflammatory properties, attributed to their ability to inhibit the formation of n-6 PUFA-derived eicosanoids (9). Resolvins and protectins generated from EPA (C20:5n-3) and DHA (C22:6n-3) display potent anti-inflammatory properties and are recognized in the resolution of inflammation (10).

Experimental studies have been performed on rats for the investigation of the prevention of hepatic IR injury by administering an n-3 PUFA-rich diet (11,12). It was shown that n-3 PUFA treatment effectively reduced hepatic steatosis and consequently attenuated hepatic IR injury in rats (11). A diet enriched with n-3 has also been shown to have a pre-conditioning effect to reduce liver IR injury in rats (12). Liver pre-conditioning against IR injury by n-3 PUFA supplementation has been reported to be mediated by the antagonistic effect of peroxisome proliferator-activated receptor alfa with the nuclear factor-kappa-B-controlled transcription of pro-inflammatory mediators (13). A recent study performed on 66 liver transplant patients showed that post-transplant parenteral nutritional support combined with n-3 fatty acids can significantly improve liver injury and shorten post-transplant hospital stays (14).

Although the effect of n-3 PUFA supplementation on liver IR injury has been extensively studied, changes in endogenous PUFA levels following liver IR injury without n-3 or n-6 diet supplementation has not been investigated. The aim of the present study was to investigate changes in liver PUFA levels following warm IR injury and determine prostaglandin E2 (PGE2) levels as well as phospholipase A2 (PLA2) and cyclooxygenase (COX) activity after re-perfusion.

## Materials and methods

### Animals

All experimental protocols conducted on rats were performed in accordance with the standards established by the Institutional Animal Care and Use Committee of Akdeniz University Medical School (Antalya, Turkey). A total of 15 male Wistar rats weighing 350–450 g, aged 5–8 months were housed in stainless steel cages and were allowed free access to standard rat chow (Korkutelim Yem, Antalya, Turkey) containing 6.05% crude fat which included linoliec acid, linolenic acid, saturated fatty acids and monounsaturated fatty acids. The animals were maintained at a 12-h light/dark cycle and a constant temperature of 23±1°C at all times.

### Rat model of hepatic ischemia-reperfusion injury

Animals were fasted 12 h prior to surgery, but allowed to drink tap water *ad libitum*. Rats were anesthetized with urethane anesthesia. Urethane (Sigma-Aldrich, Steinheim, Germany) was dissolved in 0.9% NaCl and administered at 1.2 g/kg subcutaneously. A model of lobar (70%) hepatic warm ischemia was performed according to a previously described method (15,16). After shaving and disinfecting the abdomen with betadine (Kimpa Kimya, Istanbul, Turkey), a complete midline incision was made. The portal vein was exposed and vessels supplying the median and left lateral hepatic lobes were clamped for 60 min. Re-perfusion followed for 60 min via removal of the microvascular clip. The caudal and right lobes retained an intact portal and arterial blood flow, in addition to venous outflow. These lobes served as a control and also prevented intestinal congestion. The abdomen was kept closed throughout the experimental period and the body temperature was maintained by placing rats under warming lamps. Blood samples were obtained prior to and after the experiment, from the tail vein and the right ventricle, respectively. At the end of the experimental period, the liver was perfused with 0.9% NaCl injected from the left ventricle via the inferior vena cava. Tissue samples obtained from the left and median lobes of the liver accounted for IR, while dissected right lateral and caudate lobes served as non-ischemic samples. Obtained liver tissues were either snap frozen in liquid nitrogen and stored at −70°C or fixed for histological evaluation with neutral-buffered formalin (Sigma-Aldrich). The rat model of hepatic IR injury established in the present study allowed for obtaining control and IR-injured tissue from the same liver. Via this method, control and IR liver samples (n=10 each) were obtained from rats that underwent IR injury. Sham livers (n=5) were obtained from rats in which only laparotomy was performed.

### Histopathological evaluation of liver sections

Paraffin sections stained with hematoxylin and eosin (Merck, Darmstadt, Germany) were evaluated by a pathologist blinded to the experimental conditions using an Olympus 1X81 microscope (Olympus, Tokyo, Japan). 20 high-power fields (magnification, ×200) were evaluated in all sections for congestion, intracellular edema and necrosis as previously described (17). Congestion and intracellular edema were scored as follows: 0, none; 1, present in zone III; 2, present in zones II-III; 3, present in zones I-III. Necrosis was scored as follows: 0, none; 1, single or focal necrosis; 2, submassive necrosis; 3, massive necrosis + infarction. Total histopatho-logical score was obtained by summation of all scores given for each parameter.

### Measurement of serum alanine aminotransferase

Serum alanine aminotransferase (ALT) activity was measured via an alanine transaminase assay kit (cat no. 700260; Cayman Chemical, Ann Arbor, MI, USA). The rate of nicotinamide adenine dinucleotide (NADH) oxidation was monitored by a coupled reaction system using lactate dehydrogenase (LDH). One unit of enzyme activity was defined as the amount of enzyme that caused the oxidation of 1 *µ*mol NADH to NAD^+^ per minute at 37°C.

### Electron spray ionization mass spectrometry (ESI-MS)

Standards for AA (C20:4n-6), DGLA (C20:3n-6), EPA (C20:5n-3) and DHA (C22:6n-3) were purchased from Sigma-Aldrich (St. Louis, MO, USA). Deuterium-labeled AA-d8 internal standard (5,6,8,9,11,12,14,15-AA-d8) was obtained from Santa Cruz Biotechnology (Dallas, TX, USA). Solutions of AA, DGLA, EPA, DHA and AA-d8 standards were prepared in analytical grade methanol (Merck). An optimized multiple reaction monitoring (MRM) method was developed using ultra-fast liquid chromatography (UFLC) coupled with tandem mass spectrometry (MS/MS). A UFLC system (LC-20 AD UFLC XR; Shimadzu Corporation, Kyoto, Japan) was coupled to an LCMS-8040 triple quadrupole mass spectrometer (Shimadzu Corporation). Chromatographic separations were performed using an Inertsil high-performance liquid chromatography column (ODS-4; 2.1×100 mm; 3 *µ*m; GL Sciences Inc., Tokyo, Japan) maintained at 40°C. DHA, EPA, AA and DGLA were separated using a gradient elution with a flow rate of 0.45 ml/min. Mobile phase solvent A was 10 mM ammonium acetate (Sigma-Aldrich) in water and solvent B was acetonitrile (Sigma-Aldrich). The gradient program was solvent B, 70% (0 min), 90% (3 min), 100% (3.01–4 min) and 70% (4.01–8 min). MRM transitions and responses were automatically optimized for individual compounds in negative ion ESI mode. The m/z values for the precursor and products of AA, DHA, EPA, DGLA and AA-d8 in the negative ESI-MS mode are stated in the Results section. Responses to AA, DHA, EPA and DGLA were optimized to a linear calibration range from 100 ng/ml to 30 *µ*g/ml and a sample analysis time of 8 min.

### Sample preparation for liquid chromatography (LC)-MS/MS

Samples were prepared for LC-MS/MS analysis using previously described method with certain modifications (18,19). All tissues were weighed and homogenized in ice-cold 50 mmol/l sodium phosphate buffer (pH 7.4). Homogenates were centrifuged (10,000 xg for 15 min at 4°C) and supernatants were stored at −80°C. Briefly, in a glass test tube, 200 *µ*l tissue supernatant was added to 200 *µ*l AA-d8 internal standard solution. 1 ml acetonitrile/37% hydrochloric acid (Cayman Chemical) was added to the mixture at a 4:1 v/v ratio. Tubes were capped with re-usable teflon liner screw caps and samples were hydrolyzed by incubating at 90°C for 2 h in a heating block (VLM, Bielefeld, Germany). After cooling down to room temperature, fatty acids were extracted with 2 ml hexane. Samples were vortex-mixed for 20 sec, left at room temperature for 5 min and centrifuged at 825 x g for 1 min. The upper phase containing free fatty acids was transferred to a glass tube and evaporated at room temperature under a constant stream of nitrogen with a height-adjustable gas distribution unit (VLM). Fatty acids were dissolved in 200 *µ*l methanol-water (180:20, v/v) filtered via 0,2-*µ*m polytetrafluoroethylene syringe filters (Whatman, GE Healthcare Bio-Sciences, Pittsburgh, USA) and transferred to autosampler vials (Vertical Chromatography, Nonthaburi, Thailand).

### Measurement of total PLA2 in the liver

The activity of liver PLA2 was measured via a PLA2 assay kit (cat no. ab133090; Abcam, Cambridge, MA, USA). Liver tissues were weighed and homogenized in ice-cold 50 mmol/l sodium phosphate buffer (pH 7.4) containing 1 mM EDTA. Homogenates were centrifuged (10,000 xg for 15 min at 4°C) and supernatants were stored at −80°C. Prior to performing the assay, low-molecular-weight contaminants were removed from the samples using an ultra-filtration unit via centrifugation through a 10-kDa molecular mass cut-off filter (Amicon, Millipore Corporation, Bedford, MA, USA) for 30 min at 25°C. Samples were re-constituted with 50 mmol/l sodium phosphate buffer (pH 7.4) containing 1 mM EDTA. Arachidonoyl thio-PC synthetic substrate was used to detect PLA2 activity. Hydrolysis of the arachidonoyl thioester bond releases a free thiol, which was detected by 5,5′-dithiobis-(2)-nitrobenzoic acid. One unit of enzyme activity was defined as the amount of enzyme that hydrolyzed one *µ*mol of arachidonoyl thio-PC per minute at 25°C.

### Measurement of COX activity in the liver

Liver tissues were weighed and homogenized in 0.1 M ice-cold Tris-HCl buffer at pH 7.8 containing 1 mM EDTA. Tissue homogenates were centrifuged at 10,000 xg for 15 min at 4°C and supernatants were kept at −80°C until assayed. COX activity was measured using a COX activity assay kit (cat no. 760151; Cayman Chemical) according to manufacturer's instructions. The COX activity assay kit measures enzyme activity colorimetrically by monitoring the appearance of oxidized N,N,N′,N′-tetramethyl-*p*-phenylenediamine (TMPD) at 590 nm on a microplate spectrophotometer (BioTek Instruments, Inc., Winooski, VT, USA). One unit of enzyme activity was defined as the amount of enzyme that caused the oxidation of 1 nmol of TMPD per minute at 25°C.

### Determination of PGE2

PGE2 was quantified in tissue samples by a commercial enzyme immunoassay test kit (cat no. 514010; Cayman Chemical) according to manufacturer's instructions. Liver tissues were weighed and homogenized in 0.1 M ice-cold phosphate buffer at pH 7.4 containing 1 mM EDTA and 10 *µ*M indomethacin. Tissue homogenates were centrifuged at 10,000 xg for 15 min at 4°C and supernatants were kept at −80°C until assayed. Briefly, PGE2 present in the sample competes with acetylcholinesterase-labeled PGE2 antibody for binding sites on a goat polyclonal anti-mouse antibody. Following washing to remove unbound materials, a substrate solution was added to the wells to determine the bound enzyme activity. The color development was stopped, and the absorbance was read at 412 nm. The intensity of the color was inversely proportional to the concentration of PGE2 in the sample. A standard curve of absorbance values of known PGE2 standards was plotted as a function of the logarithm of PGE2 standard concentrations (pg/ml) using the GraphPad Prism Software program for windows version 5,03. (GraphPad Software Inc, La Jolla, CA, USA). PGE2 concentrations in the samples were calculated from their corresponding absorbance values via the standard curve.

### Protein measurements

Protein concentrations were measured at 595 nm by a modified Bradford assay using Coomassie Plus reagent with bovine serum albumin as a standard (Pierce Chemical Company, Rockford, IL, USA).

### Statistical analysis

Data were analyzed using Sigma Stat (version 2.03; SyStat Software, Inc., San Jose, CA, USA) statistical software for Windows, and P<0.05 was considered to indicate a statistically significant difference between values. Values are expressed as the mean ± standard deviation. Statistical analyses for each measurement are specified in the figure and table legends.

## Results

### Confirmation of IR-induced liver injury

**Figure 1:**
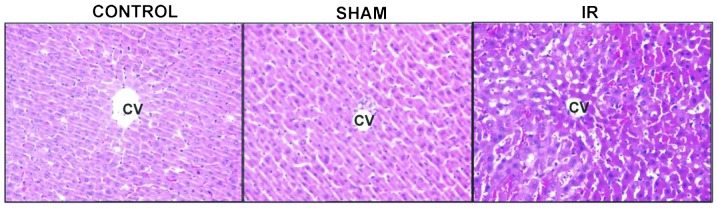
Hematoxylin and eosin staining of liver sections. Representative hepatic photomicrographs from each of the three groups (magnification, ×200). IR, ischemia/re-perfusion; CV, central vein.

Representative hepatic photomicrographs of rats from each group are shown in Fig. 1. Histopathological scores of IR-induced liver injury are listed in Table I. Intracellular edema, necrosis and total histopathological score were significantly greater (P<0.05) in the IR group compared to those in the sham and control groups. ALT levels following IR-induced liver injury are stated in Table II. Serum ALT levels were significantly increased in the IR group compared with those in the other groups, confirming the presence of hepatic injury.

### ESI-MS spectra

**Figure 2:**
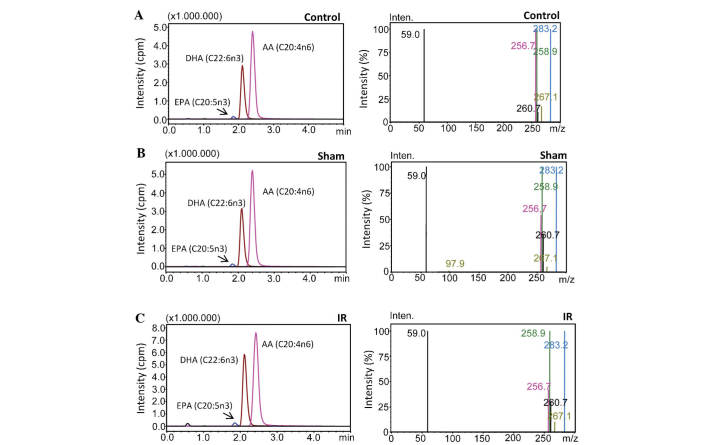
Representative (left) electron spray ionization mass spectra and (right) tandem mass spectra of (A) control (B) sham and (C) IR groups. Spectra were recorded in negative ion mode. IR, ischemia/re-perfusion; AA, arachidonic acid; EPA, eicosapentaenoic acid; DHA, docosahexaenoic acid.

The precursor and product m/z values for analyzed PUFAs were as follows: DGLA (C20:3n6), precursor m/z: 304.80, product m/z: 59.00 and 260.70; AA (C20:4n6), precursor m/z: 303.10, product m/z: 59.00 and 258.90; EPA (C20:5n3), precursor m/z: 301.10, product m/z: 59.10 and 256.70; DHA (C22:6n3), precursor m/z: 327.10, product m/z: 59.10 and 283.20; AA-d8, precursor m/z: 311.10, product m/z: 59.10 97.90 and 267.10. Fig. 2A–C shows representative negative ion mode spectra of a control, sham and IR tissue sample, respectively. As shown in Fig. 2 (left-hand panel), the retention time of EPA (C20:5n-3), DHA (C22:6n-3) and AA (C20:4n-6) was 1.869, 2.131 and 2.391 min, respectively. The right-hand panel of Fig. 2 shows tandem mass spectra obtained by collision-induced dissociation of precursor ions. The m/z values of the product ions corresponded to endogenous C20:5n3, C20:4n6, C20:3n6 and C22:6n3. The deuterium-labeled internal standard fatty acid peaks are indicated at m/z 97.9 and 267.1.

### Levels of PUFAs are increased following IR-induced liver injury

Levels of PUFAs were determined by integration of the chromatograms from LC-MS/MS analysis. Levels of PUFAs in the control, sham and IR groups are listed in Table III. Endogenous tissue levels of DGLA, AA, EPA and DHA were significantly increased following IR injury when compared to those in the control and sham groups. No significant differences in the AA/DHA and AA/EPA ratios was observed among the experimental groups.

### Total PLA2 activity is increased in liver tissue following IR-induced injury

**Figure 3:**
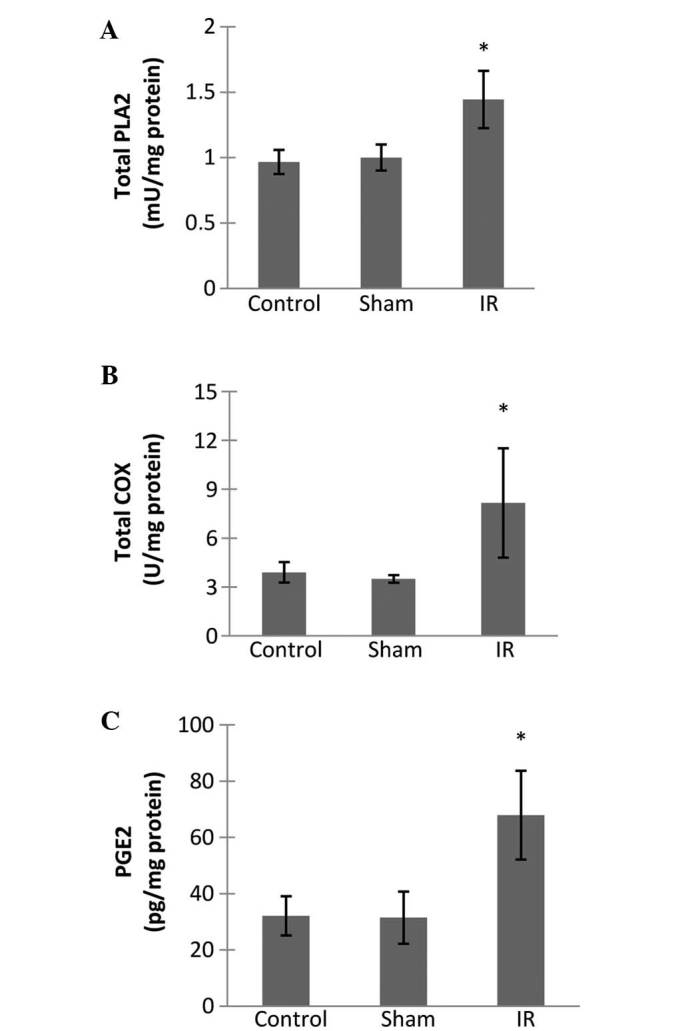
(A) Activity of total PLA2 in the liver. Statistical was performed by one-way analysis of variance and all pairwise multiple comparisons were via Tukey's test. (B) Activity of total COX in the liver. Statistical analysis was performed by Kruskal-Wallis one-way analysis of variance and all pairwise multiple comparisons were by Dunn's method. (C) Levels of PGE2 in the liver. Statistical analysis was performed by one-way analysis of variance and all pairwise multiple comparisons were via Tukey's test. Values are expressed as the mean ± standard deviation. ^*^P<0.001 vs. sham and control. IR, ischemia/re-perfusion; PLA2, phospholipase A2; COX, cyclooxygenase;.PGE2, prostaglandin E2.

Total PLA2 activity measured in IR tissue homogenates (n=10) was significantly higher compared to that in the control (n=10) and sham (n=5) groups with values of 1.44±0.22 vs. 0.97±0.09 and 1.00±0.10 mU/mg protein, respectively (Fig. 3A). No significant difference was observed between control and sham groups.

### Total COX activity is increased in the liver following IR-induced injury

Total COX activity in IR tissue homogenates (n=10) was significantly higher compared to that in the control (n=10) and sham (n=5) groups with levels of 8.16±3.36 vs. 3.90±0.63 and 3.49±0.23 U/mg protein, respectively (Fig. 3B). No significant difference was observed between control and sham groups.

### PGE2 content in the liver is increased following IR

The PGE2 content in the liver is shown in Fig. 3C. PGE2 measured in IR samples (67.91±15.81 pg/mg protein) was significantly higher compared to that in the control (32.12±6.96 pg/mg protein) and sham (31.47±9.28 pg/mg protein) groups. No significant difference was observed between control and sham groups.

## Discussion

The present study investigated changes of endogenous PUFA levels following liver IR injury in rats without n-3 or n-6 dietary supplementation. To the best of our knowledge, the present study was the first to measure endogenous DGLA (C20:3n-6), AA (C20:4n-6), DHA (C22:6n-3) and EPA (C20:5n-3) levels following liver IR injury via optimized multiple reaction monitoring using LC-MS/MS.

Clamping of the hepatic artery and portal vein (Pringle manoeuvre) is often employed to reduce excessive blood loss during liver resection (20). This procedure unavoidably leads to warm IR injury. In the present study, vessels supplying the median and left lateral hepatic lobes were clamped for 60 min and subsequent re-perfusion for 60 min via removal of the microvascular clip. Serum ALT activity was significantly increased in this model of warm liver IR. The increase in serum activity of ALT is a specific marker of liver damage (21) and thus confirms the presence of hepatic injury in the animal model used herein. Considering that ALT activity in the liver is significantly greater than that in serum, a small amount of enzyme released from tissue can cause a significant increase in circulating plasma levels of the enzyme. Histopathological evaluation of liver sections confirmed the presence of liver IR injury and was in agreement with biochemical findings of increased serum activity of ALT. As stated above, the total histopathological score of liver IR injury was obtained by summation of all scores for intracellular edema, congestion and necrosis. Histopathological evaluation revealed that intracellular edema, congestion and necrosis in IR-injured livers were greater than those in the control and sham groups; these results are reflected in the total score which was significantly higher in livers that underwent IR.

Liver AA (C20:4n-6), DGLA (C20:3n-6), EPA (C20:5n-3) and DHA (C22:6n-3) were significantly increased following IR injury compared to those in the control and sham groups. No significant difference was observed in the AA/DHA and AA/EPA ratio between the IR injury group and the control and sham groups. Competition between n-6 and n-3 fatty acids occurs in the production of eicosanoids by stereospe-cific lipid-oxidizing enzymes COX and lipoxygenase (22). Eicosanoids, derived mainly from AA (C20:4n-6), are key mediators and regulators of inflammation. These include prostaglandins (PGs), thromboxanes (TXs) and leukotrienes (LTs) (9). Elevated liver AA (C20:4n-6) levels may thus be a source of pro-aggregatory substances in liver IR injury (23). In this context, it is important to note that decreased levels of prostacyclin and increased levels of TXs and LTs are associated with abnormalities in the ratio of vasodilator to vasoconstrictor mediators in liver IR injury (24).

Eicosapentaenoic acid (C20:5n3) is a precursor of eicosanoids with a less marked inflammatory effect. Lipoxins, resolvins and protectins generated from EPA (C20:5n3) and DHA (C22:6n3) display potent anti-inflammatory properties and are recognized in the resolution of inflammation (10). Hence, increased EPA and DHA levels indicate more precursors for the synthesis of anti-inflammatory eicosanoids.

Previous studies have demonstrated a marked release of prostanoids from hepatic tissue after liver transplantation (25). Increased eicosanoid synthesis is shown to be regulated at the level of key enzymes (26). The present study has addressed changes of the local availability of these enzymes after warm liver IR injury. It was observed that total PLA2 activity measured in IR tissue homogenates was significantly higher compared to that in the control and sham groups. Accumulating evidence has revealed that PLA2 has an important role in IR injury (27,28). In fact, the PLA2 inhibitor LY329722 was shown to attenuate hepatic IR injury caused by 2-h total hepatic vascular exclusion in dogs (28). PLA2 degrades cell membrane phospholipids and has an important role in the synthesis of pro-inflammatory lipid mediators, including AA (C20:4n-6) and cytokines, during IR injury after liver transplantation (28). PLA2 comprises a large group of enzymes that include secretory PLA2 (sPLA2), cytosolic PLA2 and calcium-independent PLA2 families (29). These enzymes hydrolyze the phospholipid bond at the sn-2 position. Cytosolic PLA2 and calcium-independent PLA2 are localized inside the cell and are involved in the breakdown of intracellular membranes, whereas sPLA2 is secreted during inflammatory events (30). PLA2 accelerates the breakdown of membrane phospholipids in the liver and other organs under warm ischemia and releases free fatty acids including AA (C20:4n-6) and lysophospholipids (31). Increased total PLA2 activity observed in the experimental model of the present study may therefore explain increased levels of measured PUFAs in liver tissues. Free fatty acids released via the action of PLA2 are metabolized into PGs, TXs, LTs and platelet-activating factor (32). These lipid derivatives have pro-inflammatory and vasoconstrictive effects and contribute to post-ischemic organ dysfunction.

The activity of COX, the initial enzyme of prostaglandin synthesis, was also measured in liver tissue following IR injury. COX is the rate-limiting enzyme in the production of prostanoids from arachidonic acid. Studies have shown that the COX/prostanoid pathway is activated in hepatic diseases and liver stress reactions, including alcoholic liver disease (33), liver fibrogenesis (34), viral hepatitis C (35) and IR injury of the liver (36), causing liver damage manifested as inflammation, necrosis and fatty liver. In agreement with previous studies, the results of the present study revealed a significantly increased activity of COX following IR-induced injury of the liver compared to that in the control and sham groups, suggesting that the formation of prostanoids via the COX/prostanoid pathway also has a role in the observed tissue damage.

The present study reported significantly increased liver PGE2 levels following IR in agreement with previous studies (25). Arachidonic acid is a precursor of PGE2 synthesis and production of PGs are formed by stereospecific lipid-oxidizing enzymes. PGE2, produced during inflammatory responses, mediates a variety of innate and adaptive immune responses through four receptor sub-types (37). Each receptor functions via a distinct signaling cascade and has a unique role in a variety of disease conditions (37). In the liver, endogenous PGE2 is produced mainly by activated Kupffer cells during hepatic injury (38). Previous studies have demonstrated that endogenous as well as exogenous PGE2 is protective against liver injury caused by IR (39). This effect may be due to increased inhibition of platelet aggregation, liver perfusion or direct cytoprotection by PGE2 (40). PGE2 has also been suggested to restore liver damage through the regulation of cytokine cascades (38).

In conclusion, the present study revealed that IR-induced liver injury significantly increased the concentration of AA (C20:4n-6), DGLA (C20:3n-6), EPA (C20:5n-3) and DHA (C22:6n-3) in liver tissue specimens and had no effect on the hepatic AA/DHA and AA/EPA ratios. The observed increase of endogenous PUFA levels in the liver following IR injury was accompanied by increased activity of the key enzymes PLA2 and COX, which are involved in the production of prostanoids. The results of the present study therefore suggested that increased hydrolysis of fatty acids via PLA2 triggers the activity of COX and leads to increased PGE2 levels. Future studies evaluating agents which block the formation of eicosanoids derived from n-6 PUFAs may facilitate the development and application of treatment strategies in liver injury following IR.

## Figures and Tables

**Table I:** Histopathological scores of liver sections.

Group	Congestion	Intracellular edema	Necrosis	Total score
Sham (n=4)	0.50±0.58	0.50±0.58	0.75±0.50	1.75±0.50
Control (n=8)	1.25±0.89	0.63±0.52	0.25±0.46	2.13±1.46
IR (n=8)	2.00±1.07^a^	1.88±0.64^b^	1.88±0.84^b^	5.75±2.12^b^

Values are expressed as the mean ± standard deviation. Statistical analysis for congestion, necrosis and total score were performed by one-way analysis of variance and all pairwise multiple comparisons were performed using Tukey's test. Statistical analysis for intracellular edema was performed by Kruskal-Wallis one-way analysis of variance and all pairwise multiple comparisons by Dunn's method.

a P<0.05 vs. sham;

b P<0.05 vs. sham and control. IR, ischemia/re-perfusion.

**Table II:** Plasma activity of ALT.

Group	n	Plasma ALT (U/l)
Control	10	27.56±4.41
Sham	5	24.61±2.40
IR	10	198.25±44.56^a^

All values are expressed as the mean ± standard error of the mean. IR, ischemia/re-perfusion. Statistical analysis was performed by Kruskal-Wallis one-way analysis of variance on ranks and all pairwise multiple comparisons were via Dunn's method.

a P<0.05 vs. control and sham. ALT, alanine aminotransferase.

**Table III:** Analysis of polyunsaturated fatty acids in liver tissue.

Parameter	Control (n=10)	Sham (n=5)	IR (n=10)
DGLA (C20:3n6)	2.95±1.56	4.98±1.94	7.86±2.41^a^
AA (C20:4n6)	29.36±12.73	38.26±17.72	62.16±17.68^a^
EPA (C20:5n3)	0.86±0.53	0.80±0.13	2.27±0.87^a^
DHA (C22:6n3)	10.02±4.82	10.67±2.39	20.43±7.63^a^
AA/DHA	3.01±0.76	3.53±1.14	3.26±1.06
AA/EPA	39.07±12.83	29.39±7.86	26.32±2.57

Values are stated in mg fatty acid/g tissue protein and are expressed as the mean ± standard deviation. Statistical analyses were performed by one-way analysis of variance and all pairwise multiple comparisons via Tukey's test.

a P<0.05 compared to control and sham groups. IR, ischemia/re-perfusion; DGLA, dihomo-gamma-linolenic acid; AA, arachidonic acid; EPA, eicosapentaenoic acid; DHA, docosahexaenoic acid.
